# Superoxide Enhances the Antitumor Combination of Ad*MnSOD* Plus BCNU in Breast Cancer

**DOI:** 10.3390/cancers2010068

**Published:** 2010-02-12

**Authors:** Wenqing G. Sun, Christine J. Weydert, Yuping Zhang, Lei Yu, Jingru Liu, Douglas R. Spitz, Joseph J. Cullen, Larry W. Oberley

**Affiliations:** 1 Free Radical and Radiation Biology Program, Department of Radiation Oncology, Holden Comprehensive Cancer Center, Iowa City, IA, USA; E-Mails: wenqing-sun@uiowa.edu (W.S.); weydertc@yahoo.com (C.W.); yuping-zhang@uiowa.edu (Y.Z.); lei-yu-1@uiowa.edu (L.Y.); jingru-liu@uiowa.edu (J.L.); douglas-spitz@uiowa.edu (D.S.); 2 Department of Surgery, The University of Iowa Carver College of Medicine, Iowa City, Iowa and the VA Medical Center, Iowa City, IA, USA

**Keywords:** MnSOD, BCNU, hydrogen peroxide, superoxide, adenovirus, breast cancer

## Abstract

Overexpression of manganese superoxide dismutase (MnSOD) can sensitize a variety of cancer cell lines to many anticancer drugs. Recent work has shown that cancer cells can be sensitized to cell killing by raising peroxide levels through increased manganese superoxide dismutase (MnSOD) when combined with inhibition of peroxide removal. Here we utilize the mechanistic property of one such anticancer drug, BCNU, which inhibits glutathione reductase (GR), compromising the glutathione peroxidase system thereby inhibiting peroxide removal. The purpose of this study was to determine if anticancer modalities known to produce superoxide radicals can increase the antitumor effect of MnSOD overexpression when combined with BCNU. To enhance MnSOD, an adenoviral construct containing the cDNA for *MnSOD* (Ad*MnSOD*) was introduced into human breast cancer cell line, ZR-75-1. Ad*MnSOD* infection alone did not alter cell killing, however when GR was inhibited with either BCNU or siRNA, cytotoxicity increased. Futhermore, when the Ad*MnSOD* + BCNU treatment was combined with agents that enhance steady-state levels of superoxide (TNF-α, antimycin, adriamycin, photosensitizers, and ionizing radiation), both cell cytotoxicity and intracellular peroxide levels increased. These results suggest that the anticancer effect of Ad*MnSOD* combined with BCNU can be enhanced by agents that increase generation of superoxide.

## 1. Introduction

Reactive oxygen species (ROS), including superoxide anion (O_2_^•−^) and hydrogen peroxide (H_2_O_2_), are produced in many aerobic cellular metabolic processes. The biological effects of ROS depend on their concentration. At lower levels, ROS are involved in cell proliferation [1], differentiation [2], and signal transduction [3,4,5], while higher concentrations of ROS can cause cell death [6,7]. The steady-state intracellular concentration of ROS depends on the production and/or removal by the antioxidant system. One of the important features of antioxidant enzymes is that they are highly compartmentalized in cells. For example, manganese SOD (MnSOD) is found in the mitochondrial matrix where 75% of cellular O_2_^•−^ is generated. Cancer cells almost always express low levels of MnSOD and increasing the activity of MnSOD reverses the cancer phenotype [8,9,10,11,12]. 

The mechanism of the tumor growth suppressing effect of MnSOD has not been clearly elucidated. MnSOD catalyzes the dismutation of O_2_^•−^ to H_2_O_2_, which in turn affects downstream signal transduction pathways modulating cell proliferation [13,14]. The tumor-suppressive effect of MnSOD is supported by many studies demonstrating that overexpression of MnSOD in transformed cell lines leads to the reversion of the malignant phenotype [9,10,11,12]. In contrast, addition of pyruvate, a scavenger of H_2_O_2_, can reverse the growth inhibition of MnSOD-overexpressing cells [15]. Further evidence suggesting a role for H_2_O_2_ in modulating the cancer phenotype comes from studies combining overexpression of MnSOD with either catalase [16] or glutathione peroxidase (GPx) [17] which can reverse the inhibition of cell growth induced by MnSOD overexpression. These findings implicate H_2_O_2_ as an important mediator for the inhibition of cell growth induced by MnSOD overexpression. This increased steady-state concentration of H_2_O_2_ in the mitochondrial microenvironment following MnSOD expression is in direct proximity to many electron transport enzymes containing Fe-S centers. Interacting with these Fe-S centers can result in H_2_O_2_ and Fe^2+^ reactions leading to production of HO^•^ *via* the metal-catalyzed Haber-Weiss reaction or the production of ferryl or perferryl species [18]. Thus, there is compelling scientific evidence suggesting that the overexpression of MnSOD can sensitize cancer cells to oxidant stress [19]. 

Modification of peroxide removal mechanisms can further enhance oxidative stress downstream of O_2_^•−^ dismutation. Zhong *et al.* [20] modulated peroxide removal with two compounds that interfere with the redox buffer glutathione (GSH), an essential molecule for recycling GPx pathway by delivering either, buthionine sulfoximine (BSO), an inhibitor of glutathione synthesis, or 3-bis-chloroethyl-l-nitrosourea (BCNU) a chemotherapy drug that decomposes to form an alkylating moiety interacting with DNA as well as a carbamyolating moiety associated with the inactivation of glutathione reductase (GR) [21,22,23]. Because GR catalyzes the conversion of glutathione disulfide (GSSG) to glutathione (GSH), its loss as well as the loss of GSH will effectively reduce the peroxide-removing ability of GPx. The results of treatment with both of these molecules caused dramatic cell killing in glioma cells that stably overexpressed MnSOD [20]. Furthermore, in pre-clinical studies conducted by Weydert *et al.* in our laboratory, BCNU increased the effectiveness of AdMnSOD, dramatically reducing tumor growth and increasing survival in human oral squamous cancer [24]. 

The purpose of our present study is to determine if increased generation of superoxide radical could increase the antitumor effect of Ad*MnSOD* plus BCNU treatment. Our data demonstrates that generation of superoxide radical with antimycin, tumor necrosis factor-α, adriamycin, photodynamic action, or ionizing radiation, enhances the cytotoxicity of Ad*MnSOD* plus BCNU.

## 2. Materials and Methods

### 2.1. Cell Culture

The human breast carcinoma cell line ZR-75-1 (American Type Culture Collection) were cultured in RPMI 1640 medium with 2 mM L-glutamine adjusted to contain 1.5 g/L sodium bicarbonate, 4.5 g/L glucose, 10 mM HEPES, and 10% fetal bovine serum changing media every 3–4 days. Cells were incubated under a humidified atmosphere of 95% air/5% CO_2_ at 37 °C and passed weekly by treatment with 0.25% trypsin/0.02% EDTA. 

### 2.2. Reagents

The primary polyclonal antibodies against human MnSOD and CuZnSOD were developed in our laboratory [25]. Human glutathione peroxidase (GPx1) and glutathione reductase (GR) primary antibodies were obtained from Lab Frontier (Seoul, Korea). Horseradish peroxidase conjugated to goat anti-rabbit IgG and blocking reagents were purchased from Boehringer Mannheim (Indianapolis, IN). RPMI 1640 medium and fetal bovine serum (FBS) were purchased from HyClone (Logan, Utah). DCFH-DA and the oxidation-insensitive probe (C369) were bought from Molecular Probes (Eugene, OR). BCNU and Adriamycin were purchased from the clinical pharmacy at the University of Iowa Hospitals and Clinics. TNF-α and antimycin were purchased from Sigma Co. (Saint Louis, MO). Crystal violet and trypan blue were obtained from Fisher Scientific Co. (Pittsburgh, PA). Photofrin^TM^ (porfimer sodium) was a gift from QLT Phototherapeutics (Vancouver, British Columbia, Canada). A stock solution was made in 5% dextrose (pH 7.4), sterile filtered (0.22 μm), and stored at −20 °C. 

### 2.3. Trypan Blue Dye Exclusion Assay

The trypan blue dye exclusion was used to determine the cell viability. Twenty-four hours after treatment, cells were trypsinized and incubated with 0.2% trypan blue for 2 min at room temperature. The cells excluding the dye or stained were counted under a hemocytometer. The cell killing was indicated by the percentage of cells that were stained.

### 2.4. Adenovirus Infection

Ad*CMVMnSOD* (Ad*MnSOD*) was manufactured at the University of Iowa’s Vector Core Facility by inserting the MnSOD gene into the E1 region of an Ad5 E1/partial E3 deleted replication deficient adenoviral vector [24]. The cDNA was under the control of a CMV promoter. The MOI for all experiments were calculated from the plaque forming units (pfu), which was estimated as 1% of the total particles. ZR-75-1 cells were seeded in tissue culture plates and allowed to attach for 24 hours. Cells were then incubated with serum-free media containing adenovirus for 24 hours. Ad*LacZ* was utilized as a vector control. Adenovirus-containing medium was replaced with complete medium for an additional 24 hours before cells were harvested or treated.

### 2.5. Photofrin^TM^ Photosensitization

ZR-75-1 cells were seeded into tissue culture plates and exposed to 6 μg/mL Photofrin^TM^ in Hanks' buffer for 45 min. Cells were then irradiated with visible light (2.2 mW cm^−2^) for 2 min. After Photofrin^TM^ and light treatment, cells were allowed to recover for 6 h in full medium at 37 °C, 5% CO_2_, and 95% air in the dark.

### 2.6. Cell Homogenization and Protein Quantitation

Cells were washed three times in phosphate-buffered saline (PBS, pH 7.0, KCl 2.7 mM, KH_2_PO_4_ 1.5 mM, NaHPO_4_ 8 mM, and NaCl 136.9 mM), scraped from the dishes, and centrifuged at 82× g for 5 minutes. The supernatant was discarded and cells were resuspended in 50 mM phosphate buffer(pH 7.8). Cells were sonicated three times at maximum power, 30 seconds each time on ice using a Vibra cell cup horn sonicator (Sonics and Materials, Inc., Danbury, CT). Protein concentrations were estimated by the Bradford method and standardized with bovine serum albumin.

### 2.7. Western Blot for MnSOD Protein

Briefly, total cellular proteins were electrophoresed, transferred onto nitrocellulose membranes (Schleicher and Schuell, Keene, NH), blocked (5% milk in TTBS: 0.01 M Tris / 0.15 M NaCl buffer, pH 8.0, and 0.1% Tween 20) at room temperature for 1 h, and incubated with primary antibody (1:1000, 1 h at room temperature. After washing, blots were incubated with goat anti-rabbit IgG conjugated with horseradish peroxidase (1:1000, 1 h). Following a final wash, protein expression was visualized with chemiluminescence (ECL Plus) western blot detection solution (Amersham Pharmacia Biotech, Piscataway, NJ) exposed to X-ray film.

### 2.8. SOD Activity Gel Assay

The SOD activity gel assay is based on the inhibition of the reduction of nitroblue tetrazolium (NBT) by SOD [26]. Equal amounts of protein from different samples were subjected to 12% native polyacrylamide gels by electrophoresis in nondenaturing running buffer (pH 8.3). For SOD band visualization after electrophoresis, the gel was incubated in 2.43 mM NBT, 28 μM riboflavin-5-phosphate, 28 mM tetramethylethylenediamine, and 0.75 mM NaCN in H_2_O for 20 min under dark conditions. The gels were illuminated under a fluorescent light until achromatic SOD bands and a satisfactory blue background appeared.

### 2.9. Catalase Activity Gel Assay

The catalase activity gel assay was carried out according to the methods described by Sun *et al.* [27]. Equal amounts of protein (100 μg) from different samples were subjected to 8% native polyacrylamide gel electrophoresis in nondenaturing running buffer pH 8.3. For catalase band visualization, after electrophoresis, the gel was incubated in 0.003% hydrogen peroxide for 10 min and then stained with 2% ferric chloride and 2% potassium ferricyanide until achromatic catalase bands began to form.

### 2.10. GR Activity Gel Assay

Equal amounts of protein from different samples were subjected to 8% native polyacrylamide gels by electrophoresis in nondenaturing running buffer pH 8.3. For glutathione reductase band visualization, after electrophoresis, the gel was stained with 250 mM Tris (pH 8.0) containing 3.4 mM GSSG, 0.36 mM NADPH, 0.052 mM dichlorophenol-indophenol, and 1.1 mM 3(4,5-dimethylthiazolyl-2)-2,5-diphenyl tetrazolium bromide until blue precipitate GR bands began to form.

### 2.11. GR Activity Assay

GR activity was measured according to the methods described by Mavis and Stellwagen [28]. The GR activity assay was based on the reduction of GSSG and oxidation of NADPH by the enzymatic action of GR. The disappearance of NADPH was monitored at 340 nm as the indicator of GR activity. Working buffer contained 0.65 mol of ddH_2_O, 1.5 mL 100 mM potassium phosphate buffer with 3.4 mM EDTA (pH 7.6), 0.1 mL 30 mM GSSG, 0.35 mL 0.8 mM β-NADPH, and 0.3 mL 1.0% bovine serum albumin. At room temperature, the absorbance at 340 nm was monitored until constant then equal amount of cell extracts (500 μg/100 μL) were added in working buffer, and the decrease in absorbance in 340 nm for 3 minutes was immediately recorded. One unit of GR is defined as the amount of enzyme required to reduce 1.0 μmol of GSSG per minute at pH 7.6 at 25 °C.

### 2.12. Detection of ROS by Plate Reader

ZR-75-1 cells were plated in 24-well microtiter plates at a density of 2 × 10^4^ cells/well for 24 hours. After different treatments, medium was removed, and the cells were washed with serum-free medium twice. A solution of either 10 μM DCFH-DA (1194) or 10 μM oxidation-insensitive DCFH-DA probe (C369) in serum free media was then added for 30 min. The cells were washed with serum-free media once. SDS (0.5%) was added to lyse the cells. Immediately after lysing the cells, fluorescence was measured using a SPECTRAFluor Plus spectrofluorimeter (excitation wavelength 485 nm, emission emission wavelength 530 nm for both probes).

### 2.13. Detection of ROS by Confocal Microscopy

DCFH staining with confocal microscopy was also used to measure intracellular ROS levels. ZR-75-1 cells were seeded at a density of 3 × 10^5^ cells/ well in slide-chambers. After different treatments, media was removed, either 10 μM DCFH-DA or 10 μM oxidation-insensitive DCFH-DA probe was added for 30 min in the dark, then washed with Hank’s buffer. Cells were fixed, and fluorescence was observed by confocal microscopy at 530/485 nm for both probes. 

### 2.14. Transfection of siRNA

GR-siRNA were designed and manufactured by Ambion (Austin, TX). The target sequences for GR-siRNA were 5'-AAA GGG GTA AAT TCA ATT GGC GT-3' (forward), 5'-AAA AAC GCC AAT TGA ATT TAC CC-3' (reverse, NM 000637) respectively. SiNeg (siRNAs with sequences that do not target any gene product) was used to determine the transfection efficiency and to control for the effects of siRNA delivery. ZR-75-1 cells were transfected with siRNA (20 pmole/2 × 10^5^ cells by Lipofectamine 2000, Invitrogen) for 24 hours. The cells were incubated in full media for the indicated times prior to further experiments.

### 2.15. RT-PCR Analysis

Total RNA was isolated from cells using the RNeasy Mini Kit (QIAGEN, Valencia, CA). cDNA synthesis was performed with a High-Capacity cDNA Archive Kit (Applied Biosystems, Foster City, CA). Real time PCR was conducted using an ABI PRISM 7000 Sequence Detection System (Applied Biosystems), and the PCR amplification was then detected with the SYBR Green I Nucleic Acid gel stain (Cambrex Bio Science Rockland, Inc., Rockland, ME). The housekeeping gene 18S was used as an endogenous control.

### 2.16. Statistics

Mean values and standard errors were determined using the Microsoft Excel program. The ANOVA-Tukey test was used to compare the differences between two groups. In all cases, the statistical significance of differences between the two variants was determined at a level of P < 0.05. All the data presented are from the average of at least three independent experiments. All blots or activity gels were repeated at least twice to ensure reproducibility. 

## 3. Results

Previous work in our laboratory has demonstrated that adenovirus MnSOD cDNA transfection increases RNA, protein, and enzymatic activity of MnSOD [25,29]. These increases are both dose and time dependent. In the present study, Ad*MnSOD* (100 MOI) infection increased MnSOD immunoreactive protein and activity in ZR-75-1 cells which peaked at 48 hours after transfection persisted for more than 96 hours (data not shown).

### 3.1. Determination of Effective BCNU Treatment Concentration

**Figure 1:**
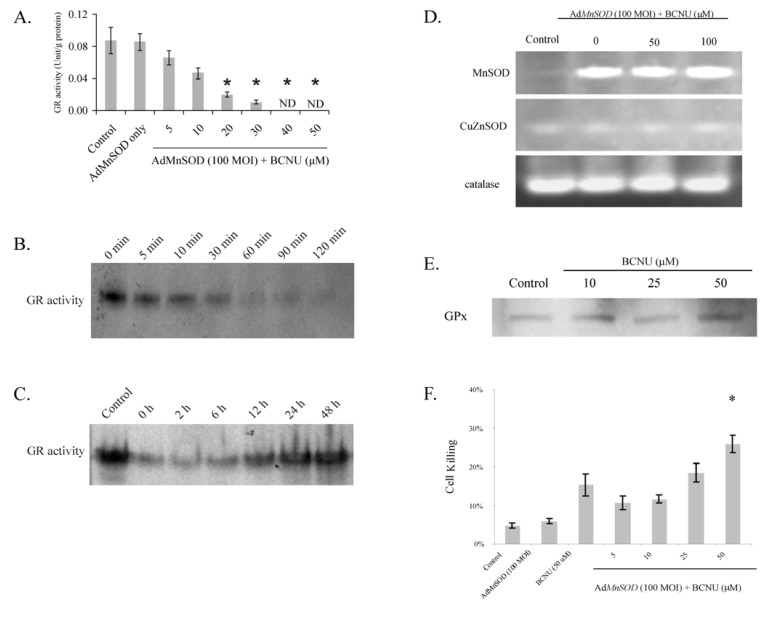
MnSOD overexpression sensitized cells to BCNU whileBCNU inhibited glutathione reductase (GR) activity in a dose-dependent manner. ZR-75-1 cells were infected with Ad*MnSOD* (100 MOI) and treated with BCNU (0–50 μM). GR activity was measured by activity gel assay or GR spectrophotometric activity assay.

In cells infected with Ad*MnSOD*, BCNU inhibited GR in both a dose- and time-dependent manner (Figure 1A). Treatment with BCNU caused a dose-dependent decrease in GR activity (5–30 μM) decreasing GR activity ≧90% at higher concentrations (Figure 1A). The inhibition of GR activity was also time-dependent as determined by activity gel analysis. Further characterization of an appropriate dosing regimen was determined over a 2 h time frame where inhibition of GR became significant after treating cells with BCNU (25 μM) for 60 minutes (Figure 1B). Moreover, the activity of GR increased gradually over time up to 48 hours after removal of BCNU whereupon the GR activity came back to control levels (Figure 1C). BCNU inhibited GR in a dose and time dependent manner. However, BCNU treatment did not affect the enzymatic activity of other major antioxidant proteins, including MnSOD, CuZnSOD and catalase (Figure 1D). Moreover, Ad*MnSOD* plus BCNU did not greatly affect the levels of GPx immunoreactive protein (Figure 1E). Finally, MnSOD overexpression sensitized cells to BCNU-induced cytotoxicity. Following transduction with Ad*MnSOD*, ZR-75-1 cells were treated with various concentrations of BCNU for 24 hours. Measuring cell killing by trypan blue dye exclusion assay, demonstrated that Ad*MnSOD* plus BCNU increased cell killing when compared to Ad*MnSOD* alone or BCNU alone (Figure 1F). Cell death was 6% with Ad*MnSOD* (100 MOI) and increased to 16% with BCNU (50 μM). The combination of Ad*MnSOD* with BCNU in ZR-75-1 cells increased cell killing to 28% (Means, P < 0.05 vs Ad*MnSOD* alone, n = 3).

(A). GR activity assay. There was a dose- dependent decrease of GR activity after BCNU treatment in the presence of Ad*MnSOD*. BCNU (40 μM or greater) resulted in nondetectable levels of GR activity. Means ± SEM, *p < 0.005 versus no treatment, n = 3; (B). GR activity was inhibited by BCNU and recovered in a time-dependent manner. ZR-75-1 cells were infected with Ad*MnSOD* (100 MOI) and treated with BCNU (25 μM) for the indicated times and GR activity was measured by activity gel assay. GR activity began to decrease after 5 minutes of BCNU (25 μM). GR activity was undetectable at 90 and 120 minutes. Experiments were repeated at least three times with similar results; (C). After 6 hours of BCNU (25 μM), there was a time-dependent recovery of GR activity; at 48 hours, the GR activity came back to control level. Experiments were repeated at least three times with similar results; (D). BCNU treatment did not affect levels of the antioxidant enzymes MnSOD, CuZnSOD and catalase in ZR-75-1 cells. The enzymatic activities of MnSOD, CuZnSOD and catalase were determined by activity gel assay. There were no significant changes in MnSOD, CuZnSOD, GPx, and catalase levels after treatment with BCNU for 60 minutes. Experiments were repeated at least three times with similar results; (E). BCNU treatment for 60 minutesdid not affect levels of GPx as determined by western blot. Experiments were repeated at least three times with similar results; (F). Ad*MnSOD* overexpression sensitized cells to BCNU. ZR-75-1 cells were infected with Ad*MnSOD* (100 MOI) and treated with different concentrations of BCNU (5–50 μM) for 60 minutes. Cell death was measured by the trypan blue dye exclusion assay. MnSOD overexpression plus BCNU resulted in significant more cell killing than BCNU alone or cells infected with Ad*MnSOD* alone. Means ± SEM, *p < 0.005 Ad*MnSOD* plus BCNU versus BCNU alone, n = 3.

### 3.2. MnSOD Overexpression Sensitizes Cells to siRNA to GR-induced Cytotoxicity

**Figure 2:**
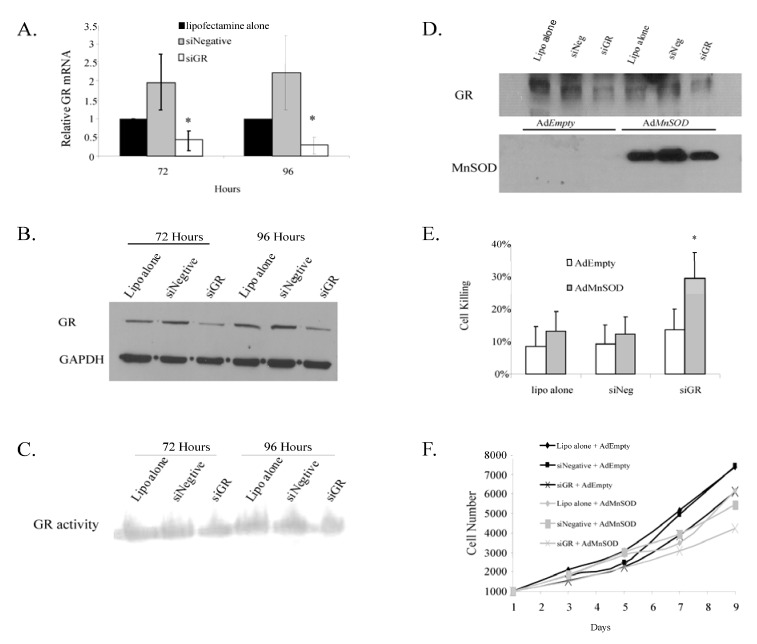
siRNA targeting the GR sequence inhibited GR mRNA, protein and activity levels and decreased cell growth and increased cell killing in ZR-75-1 cells. The cells were seeded at a density of 2.5 × 105 cells/well in 12-well plates overnight, transfected with 6 μg siRNA for 24 hours, recovered in full media for the indicated time.

Besides inhibition of GR by a carbamoylating effect, BCNU also causes DNA or RNA crosslinking *via* an alkylating effect. To try to exclude the potential alkylating effect of BCNU from our results we designed small interfering RNA (siRNA) targeted to the GR sequence (siGR) as a specific means of inhibiting GR. The siGR oligos were transferred into ZR-75-1 cells. Figure 2 demonstrates that siGR inhibited GR at the mRNA, protein and enzymatic activity levels. GR mRNA was significantly lowered after infection for 72 and 96 hours (Figure 2A), GR protein was decreased by more than 50% (Figure 2B), and GR activity was also significantly decreased (Figure 2C). Cells treated with both siGR and Ad*MnSOD* demonstrated both a decline in GR protein and an increase in MnSOD protein levels (Figure 2D). In addition, siGR and Ad*MnSOD* significantly increased cell killing to 35% (Figure 2E). In contrast, siGR plus Ad*Empty* resulted in a 13% cell killing (Figure 2E). Furthermore, siGR plus Ad*MnSOD* prolonged cell doubling time in ZR-75-1 cells from 2.8 ± 0.1 days to 3.8 ± 0.1 days (P < 0.05, Means ± SEM, n = 4) (Figure 2F).

(A). The levels of GR mRNA, as determined by real time RT-PCR, decreased significantly with si RNA targeted to GR. * p < 0.05 (siGR *vs.* lipo alone, or siNeg), n = 3; (B). Protein levels of GR were decreased as determined by western blot. For western blot of GR, 15 μg protein per well was used. Experiments were repeated at least three times with similar results. Representative results are shown; (C). GR enzymatic activity was determined by activity gel assay. For the activity gel assay 200 μg of cellular protein per well was used for detection. GR activity levels began to be inhibited 72 hours after transfection and became more significant at 96 hours. Experiments were repeated at least three times with similar results. Representative results are shown; (D). Cells were transfected by lipofectamine alone (lipo alone, lanes 1, 4), siNeg (lanes 2, 5), or siRNA to GR (siGR, lanes 3, 6) for 24 hours, recovered in full media for one day, then transfected with or Ad*Empty* (100 MOI, lanes 1–3) or Ad*MnSOD* (100 MOI, lanes 4–6) for another 24 hours and recovered for 24 hours. Western analysis demonstrates a decrease in GR protein in cells transfected with siGR and an increase in MnSOD protein in cells infected with the Ad*MnSOD* vector in ZR-75-1 cells. Experiments were repeated at least three times with similar results. Representative results are shown; (E). Cell killing was determined by trypan blue dye exclusion assay. MnSOD overexpression did not increase cell killing in cells treated by lipofectamine alone or siNeg. However siGR plus Ad*MnSOD* significantly increased cell killing compared to siGR plus AdEmpty. *p < 0.05, n = 3; (F). Cell proliferation was determined by growth curve analysis. Ad*Empty +* siGR, AdMnSOD + lipofectamine, or AdM*nSOD* + siNeg slightly delayed cell growth, while the combination of Ad*MnSOD* + siGR significantly prolonged cell doubling time. Means ± SEM, n = 4.

### 3.3. Increasing Superoxide Production Enhances Peroxide Induced Cell Toxicity: AdMnSOD Plus BCNU Sensitized Cells to Antimycin

**Figure 3:**
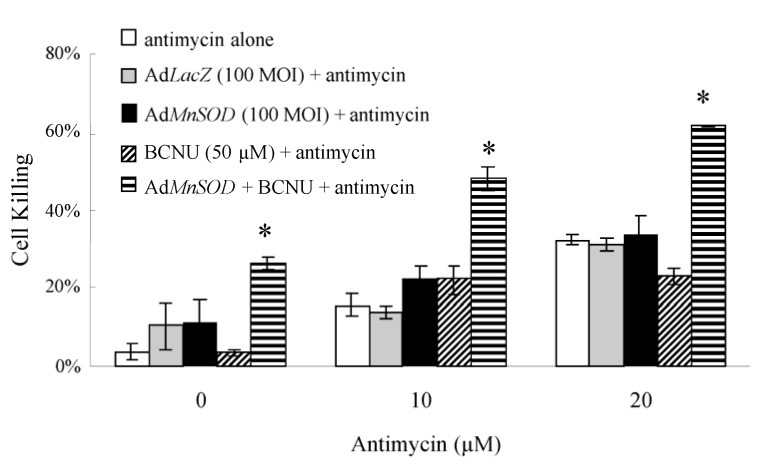
Ad*MnSOD* infection plus BCNU sensitized cells to antimycin. ZR-75-1 cells were infected with Ad*LacZ* (100 MOI) or Ad*MnSOD* (100 MOI), treated with BCNU (50 μM) for 2 hours, and then treated with antimycin (0, 10, 20 μM) for 24 hours. Cell killing was determined by trypan blue dye exclusion assay. Ad*MnSOD* plus BCNU sensitized cells to the cytotoxic effect of antimycin. For example, antimycin (20 μM) alone resulted in 30% cell killing, while the combination of Ad*MnSOD* plus BCNU plus antimycin increased cell killing to >60%. Means ± SEM, *p < 0.005 Ad*MnSOD* plus BCNU plus antimycin versus all other groups, n = 3.

Antimycin inhibits the electron transport chain by competitively binding to cytochrome b and displacing coenzyme Q resulting in electron leak at complex I and II. [30]. The combination of MnSOD, BCNU, and antimycin increased cell killing compared to any combinations of the other agents (Figure 3). For example, antimycin (20 μM) increased cell killing to 30% while the combination of Ad*MnSOD*, BCNU and antimycin increased cell killing to >60%. Other combinations including Ad*LacZ* plus antimycin, Ad*MnSOD* plus antimycin without BCNU, or BCNU plus antimycin without MnSOD overexpression did not enhance the cytotoxicity of antimycin alone.

#### 3.3.1. AdMnSOD plus BCNU Sensitized Cells to TNF-α

**Figure 4:**
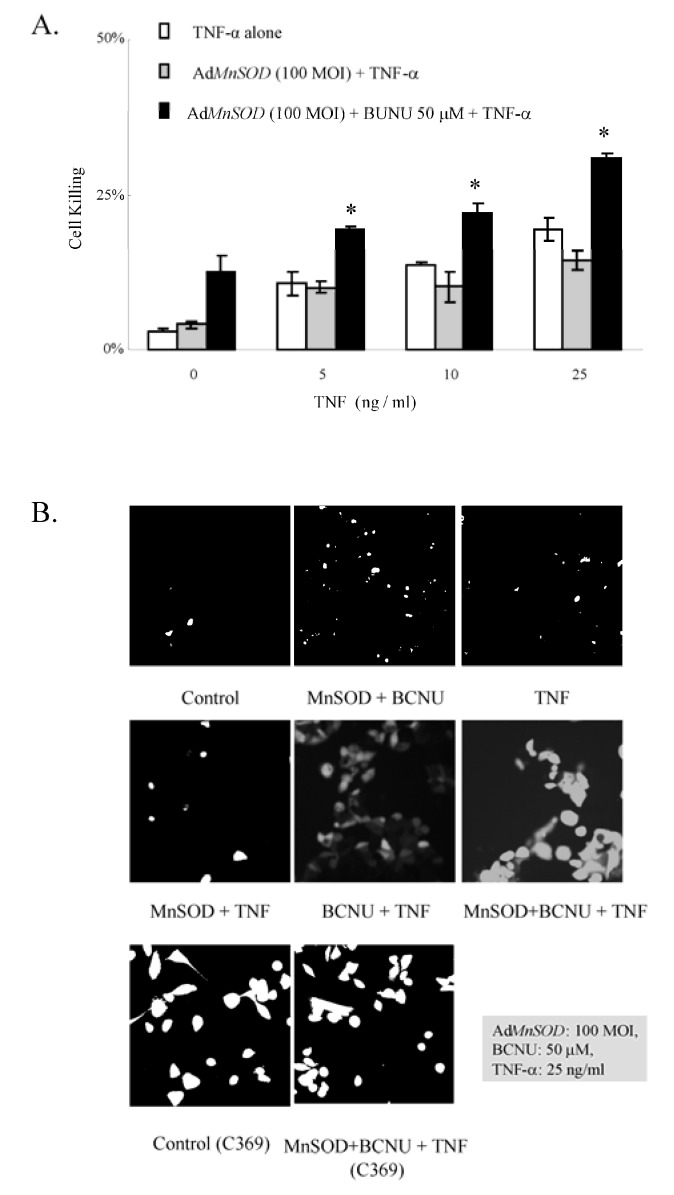
Ad*MnSOD* plus BCNU increased TNF-α-induced cytotoxicity and ROS levels.

TNF-α is a cytokine produced by activated macrophages. The effects of TNF-α on cells include DNA fragmentation [31] and oxidative damage such as lipid peroxidation [32]. Antioxidants block the cytotoxic effect of TNF-α [33] and MnSOD is sufficient for cellular resistance to the cytotoxicity of TNF-α [34], suggesting that the generation of O_2_^•−^ in mitochondria may contribute to TNF-α induced tumor cell killing. MnSOD plus BCNU sensitized cells to the cytotoxicity of TNF-α (25 ng/mL) increased cell killing to 20% cell killing as measured by the trypan blue dye exclusion assay. AdMnSOD decreased the cell killing induced by TNF-α to 12%, while the combination of AdMnSOD plus BCNU increased the cell killing induced by TNF-α to 33% (Figure 4A). The increase in cell killing correlated with ROS accumulation in the cells as AdMnSOD infection plus BCNU significantly increased ROS levels induced by TNF-α as measured by DCFH oxidation (Figure 4B). Fluorescence was unchanged between control cells and cells treated with AdMnSOD plus BCNU plus TNF-α when the oxidation-insensitive DCFH-DA probe (C369) was used (Figure 4B).

(A). ZR-75-1 cells were infected with Ad*MnSOD* (100 MOI) treated with BCNU (50 μM) for 2 hours, and treated with TNF-α (0, 5, 10, 25 ng/mL) for 48 hours. Cell killing was determined by trypan blue dye exclusion assay. Ad*MnSOD* plus BCNU plus TNF-α had the greatest cell killing effect. Means ± SEM, *p < 0.005 *versus* TNF-α alone, Ad*MnSOD* plus TNF-α. n= 3; (B). To determine ROS levels, DCFH-DA (10 μM) was added for 30 min and fluorescence determined at 530/485 nm. Control cells, cells treated with TNF-α alone, cells treated with Ad*MnSOD* plus BCNU, or cells treated with Ad*MnSOD* plus TNF-α all demonstrated relatively faint fluorescence. However, cells treated with the combination of Ad*MnSOD* plus BCNU plus TNF-α resulted in a large increase in the fluorescence intensity. In addition, the oxidation-insensitive fluorescence probe C369 was also used in controls cells and cells treated with the Ad*MnSOD* plus BCNU plus TNF-α combination demonstrating no significant differences between the C369 fluorescence intensity in these two samples. Experiments were repeated at least three times with similar results.

#### 3.3.2. AdMnSOD plus BCNU Sensitized Cells to Adriamycin

**Figure 5:**
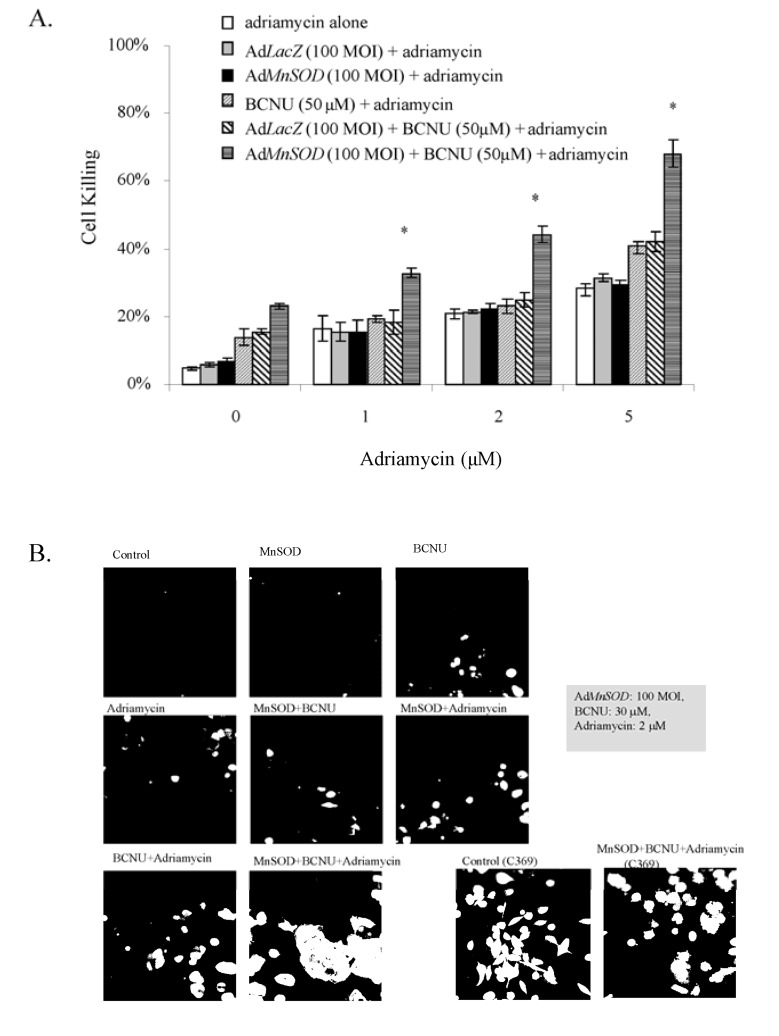
Ad*MnSOD* infection plus BCNU plus adriamycin increased cell killing.

Adriamycin is a quinone containing anti-tumor antibiotic [35]. It is electron-affinic and the acceptance of one electron causes adriamycin to be reduced to the adriamycin free radical semi-quinone [35]. This semi-quinone free radical not only induces DNA damage by itself, but also redox cycles with O2 to produce O_2_^•−^ [36]. MnSOD plus BCNU sensitized cells to the cytotoxicity of adriamycin (Figure 5A). The cytotoxicity of adriamycin in MnSOD plus BCNU-pretreated cells increased more than two-fold compared to cells treated with adriamycin alone (Figure 5A). In addition, there was an increase in DCFH oxidation when adriamycin (2 μM) was added into the cells pretreated with AdMnSOD plus BCNU (50 μM) (Figure 5B). Once again, oxidation of the probe was unchanged between control cells and cells treated with AdMnSOD plus BCNU plus adriamycin when the oxidation-insensitive DCF-DA probe (C369) was used (Figure 5B).

#### 3.3.3. AdMnSOD Plus BCNU Sensitized Cells to Photodynamic Therapy (PDT)

PDT is an effective anticancer treatment modality for esophageal, lung, and bladder cancers. Moreover, numerous clinical trials have investigated the effectiveness of PDT in the treatment of breast cancer [37,38]. The treatment consists of a systemic administration of a photosensitizer followed by illumination with a laser light. The photochemical reaction produces numerous forms of reactive oxygen, such as singlet oxygen (^1^O_2_), O_2_^•−^, H_2_O_2_, and HO^•^ [39]. After illumination of Photofrin^TM^-bearing cells, there was a 7-fold elevation of O_2_^•−^ generation [40]. SOD mimetics, beta-carotene, and dimethyl sulfoxide were protective following Photofrin^TM^-mediated PDT in mice [41]. Inhibition of SOD activity in tumor cells by a MnSOD inhibitor produced synergistic antitumor effects when combined with PDT in various tumor cell lines [42]. 

**Figure 6:**
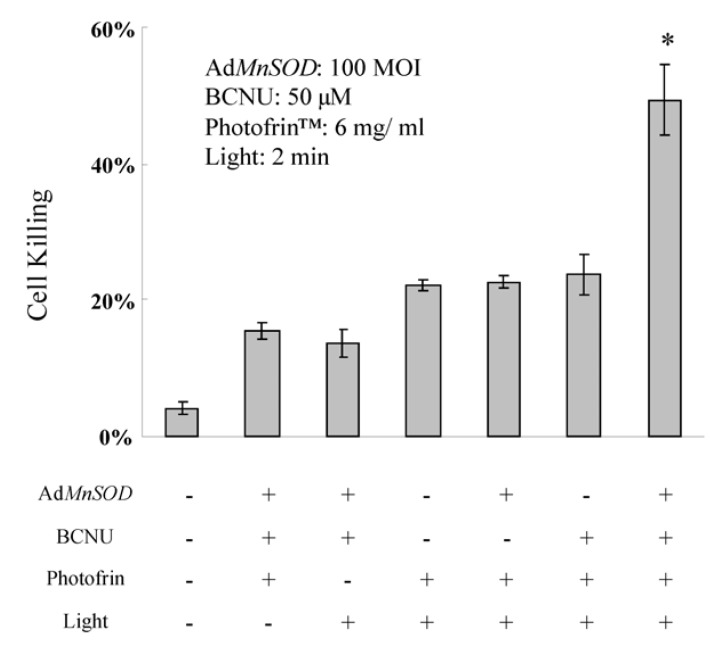
Ad*MnSOD* infection plus BCNU sensitized cells to photodynamic therapy. ZR-75-1 cells were infected Ad*MnSOD* (100 MOI) for 24 hours, recovered in full media without Ad*MnSOD* for another 24 hours, treated with BCNU (50 μM), and Photofrin^TM^ (6 mg/mL) for 1 hour. Following treatments, the cells were irradiated with visible light for 2 min. After 6 hours of incubation in the dark, cell killing was determined by trypan blue dye exclusion assay. Ad*MnSOD* plus BCNU sensitized cells to the cytotoxic effect of Photofrin^TM^ photodynamic therapy. Means ± SEM *p < 0.005, Ad*MnSOD* plus BCNU plus photodynamic therapy versus all other groups, n = 3.

MnSOD-overexpression cells were treated with BCNU (50 μM) and Photofrin^TM^ (6 mg/mL) for 1 hour and then irradiated with visible light for 2 min. After 6 hour incubation in the dark, cell killing was determined by trypan blue dye exclusion assay. Photofrin^TM^-PDA treatment alone caused a cell killing of 25% while pretreatment with Ad*MnSOD* plus BCNU increased the cell killing induced by Photofrin^TM^-PDA to 55% (Figure 6). 

(A). ZR-75-1 cells were infected with Ad*MnSOD* (100 MOI) for 24 hours, recovered in full media without Ad*MnSOD* for another 24 hours, treated with BCNU (50 μM) for 2 hours, and then treated with adriamycin (0–5 μM) for 24 h. Cell killing was determined by trypan blue dye exclusion assay. Ad*MnSOD* plus BCNU sensitized cells to the cytotoxic effect of adriamycin. *p < 0.005 Ad*MnSOD* plus BCNU plus adriamycin versus all other groups, n = 3; (B). ZR-75-1 cells were treated as described in 5A and then DCFH-DA (10 μM) was added for 30 min and washed out. Flurorescence was observed at 530/485 nm. There was a significant increase in fluorescence after treating cells with the combination of Ad*MnSOD*, BCNU, and adriamycin. The oxidation-insensitive fluorescence probe C369 was also used in control cells and cells treated by MnSOD, BCNU, and adriamycin which did not increase the fluorescence intensity of the C369 probe. Experiments were repeated at least three times with similar results. Representative results are shown.

#### 3.3.4. AdMnSOD Infection plus BCNU Sensitized Cells to Ionizing Radiation

**Figure 7:**
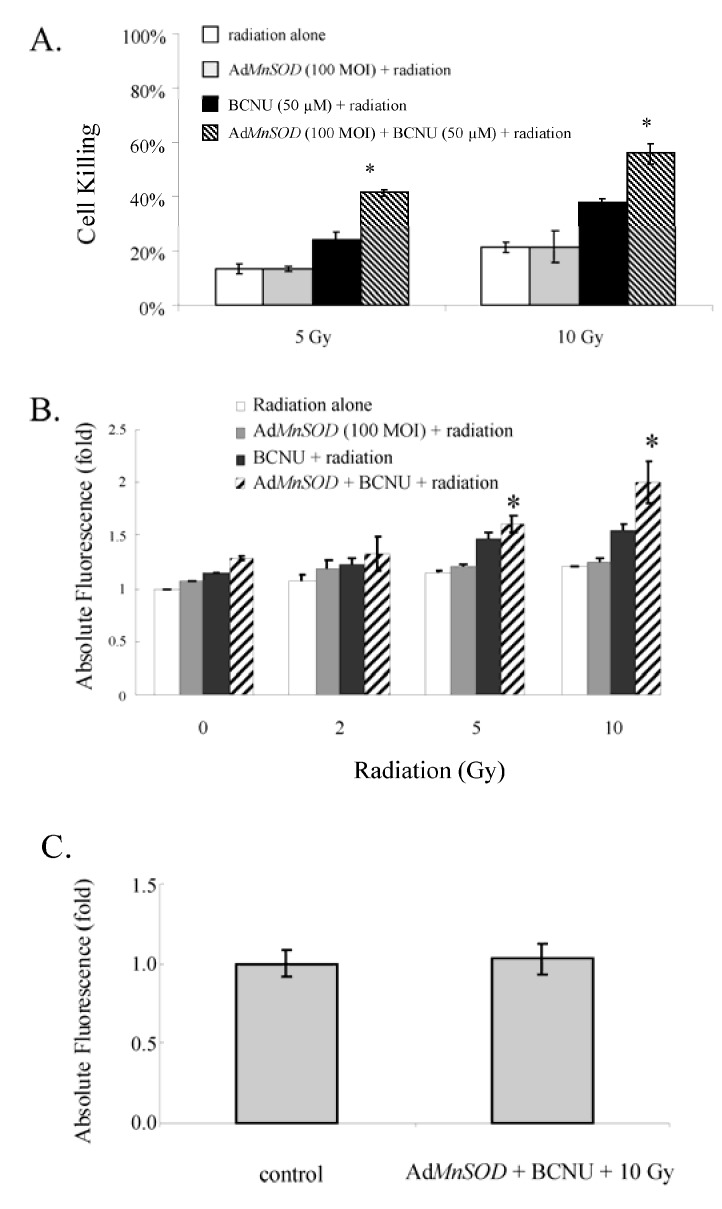
MnSOD-overexpression plus BCNU sensitized cells to radiation and increased ROS accumulation.

Upon exposure to ionizing radiation, various ROS, including O_2_^•−^, H_2_O_2_, and HO^•^ are produced. Ad*MnSOD*-infected ZR-75-1 cells were treated with BCNU (50 μM) for 2 hours and then irradiated with 2, 5, or 10 Gy. After incubation in full media for 24 hours, cell killing was determined by the trypan blue dye exclusion assay. Ad*MnSOD* or BCNU alone did not sensitize cells to the cytotoxic effect of ionizing radiation. However, Ad*MnSOD* (100 MOI) plus BCNU (50 μM) increased radiation-induced cytotoxicity by more than 2 fold (Figure 7A). The production of ROS by ionizing radiation in cells was measured by DCFH fluorescence. Fluorescence intensity was unchanged in cells irradiated at 2, 5, and 10 Gy only compared to untreated cells. In cells pretreated with Ad*MnSOD* plus BCNU, fluorescence intensity increased to 1.5- and 2-fold at 5 and 10 Gy, respectively (Figure 7B). Furthermore, there was no increase in the intensity of fluorescence detected by the oxidation-insensitive probe (C369) between the control cells and the cells treated with MnSOD, BCNU and 10 Gy radiation, indicating no change in the esterase activity or drug efflux (Figure 7C).

(A). ZR-75-1 cells were infected with Ad*MnSOD* (100 MOI) for 24 hours, recovered in full media without Ad*MnSOD* for another 24 hours, treated with BCNU (50 μM) for 2 hours, and then irradiated with either 5, or 10 Gy. After incubation in full media for 24 hours, cell killing was determined by trypan blue dye exclusion assay. Ad*MnSOD* plus BCNU sensitized cells to the cytotoxic effect of irradiation. *p < 0.005 versus radiation alone, Ad*MnSOD* plus radiation, BCNU plus radiation, n = 3; (B). ZR-75-1 cells were treated as described and 24 hours after radiation, the media was removed and the prooxidant sensitive probe DCFH-DA (10 μM) was added for 15 min and cells harvested. Fluorescence was measured at 530/485 nm. * p < 0.05 versus radiation alone, Ad*MnSOD* plus radiation, BCNU plus radiation, n = 3; (C). ZR-75-1 cells were treated as described and 24 hours after radiation, the media was removed and the oxidant-insensitive DCF-DA probe (C369) (10 μM) was added for 15 min and cells harvested. Fluorescence was measured at 530/485 nm.

## 4. Discussion

Previous work in our lab has shown that when MnSOD overexpression is combined with inhibitors of hydroperoxide removal increases cancer cell cytotoxicity in contrast to the non-cytotoxic tumor suppressive effect of MnSOD overexpression alone [24]. We hypothesize that elevating MnSOD levels in cancer cells enhances the conversion of O_2_^•−^ to H_2_O_2_. Furthermore, inhibiting the glutathione peroxidase system further raises peroxide levels by inhibiting the removal of H_2_O_2_, which contributes to the cell killing. The aim of our present study was to extend these studies by increasing production of O_2_^•−^ and subsequently sensitizing cells to the cytotoxic effect of MnSOD plus BCNU. BCNU inhibited GR activity and resulted in a gradual return of GR activity. Only GR was inhibited by BCNU, while other enzymes, such as MnSOD, CuZnSOD, and catalase were not affected. The different modalities that produce O_2_^•−^ (antimycin, TNF-α, adriamycin, Photofrin^TM^-PDA, ionizing radiation) further increased ROS levels in cells when added to the Ad*MnSOD* plus BCNU treatment. The increased ROS levels correlated with increased cell killing. The results from this study showed that addition of exogenous superoxide can enhance the antitumor effect of MnSOD plus BCNU.

Besides inhibition of GR *via* a carbamoylating effect, BCNU also causes DNA or RNA crosslinking *via* an alkylating effect. Thus, the dual effect of BCNU on enhanced cell killing may be due to the alkylating effect, instead of the inhibition of GR. In support of the carbamoylating effect of BCNU and subsequent inhibition of GR, Nathan and Cohn [43] demonstrated that BCNU could enhance the antitumor effect of H_2_O_2_. Secondly, pyruvate, a peroxide scavenger, reduced cell killing by BCNU in MnSOD-overexpressing cell lines [20]. Finally, our present study used siRNA to inhibit GR. Infection of cells with Ad*MnSOD* plus siRNA directed to GR resulted in similar results obtained with Ad*MnSOD* plus BCNU, suggesting that GR inhibition caused the killing effects of BCNU with MnSOD. Thus, H_2_O_2_ appears to be a pivotal mediator in the cell killing induced by MnSOD.

Although antimycin, TNF-α, and photodynamic therapy are not used in the treatment of breast cancer, adriamycin and ionizing radiation are extensively used. Adriamycin is a quinone containing anti-tumor antibiotic that is reduced to the adriamycin free radical semi-quinone [35] which then redox cycles with O_2_ to produce O_2_^•−^ [36]. After ionizing radiation, MnSOD protein increased in a biphasic manner [44], while overexpression of MnSOD reduces the levels of irradiation-induced inflammatory cytokines [45], and reverses radiation-induced normal cell cytotoxicty [46]. Our current study demonstrates that MnSOD overexpression can have the opposite effect on cancer cells resulting in increased cell killing, if peroxide removal is inhibited. Our present study also correlates well with recent work from our laboratory demonstrating that Ad*MnSOD* plus BCNU sensitized MB231 breast cells to the cytotoxicity of adriamycin or radiation *in vitro* and also inhibited tumor growth and prolonged survival *in vivo* [47]. Also, if we increase the levels of the substrate for MnSOD (O_2_^•−^), we can produce more product (H_2_O_2_) and thus further enhance cytotoxicity. Increased superoxide radical production can increase Ad*MnSOD* plus BCNU-induced cytotoxicity which may lead to an effective combination in the treatment of breast cancer.

## 5. Conclusions

MnSOD overexpression inhibits breast cancer cell growth. An adenoviral construct containing the cDNA for *MnSOD* (Ad*MnSOD*) was introduced into human breast cancer cell line, ZR-75-1. Ad*MnSOD* infection alone did not alter cell killing, however when glutathione reductase was inhibited with either 3-bis-chloroethyl-l-nitrosourea (BCNU) or siRNA to glutathione reductase, cytotoxicity increased. Futhermore, when the Ad*MnSOD* + BCNU treatment was combined with agents that enhance steady-state levels of superoxide (TNF-α, antimycin, adriamycin, photosensitizers, and ionizing radiation), both cell cytotoxicity and intracellular peroxide levels increased. These results suggest that the anticancer effect of Ad*MnSOD* combined with BCNU can be enhanced by agents that increase generation of superoxide.
